# Hybrid Distractor for Continuous Mandibular Distraction Osteogenesis

**DOI:** 10.3390/bioengineering9120732

**Published:** 2022-11-28

**Authors:** Shahrokh Hatefi, Javad Alizargar, Yimesker Yihun, Milad Etemadi Sh, Nan-Chen Hsieh, Khaled Abou-El-Hossein

**Affiliations:** 1 Ultra-High Precision Manufacturing Laboratory, Department of Mechatronics Engineering, Faculty of Engineering, The Built Environment and Technology, Nelson Mandela University, Port Elizabeth 6000, South Africa; 2 Research Center for Healthcare Industry Innovation, National Taipei University of Nursing and Health Sciences, Taipei City 112, Taiwan; 3 School of Nursing, National Taipei University of Nursing and Health Sciences, Taipei City 112, Taiwan; 4 Robotics and Control Laboratory, Mechanical Engineering Department, Wichita State University, Wichita, KS 67260, USA; 5 Department of Oral and Maxillofacial Surgery, Dental Implants Research Center, Dental Research Institute, School of Dentistry, Isfahan University of Medical Sciences, Isfahan 81746-73461, Iran; 6 Department of Information Management, National Taipei University of Nursing and Health Sciences, Taipei City 112, Taiwan

**Keywords:** medical device, implanted active distractor, continuous distraction osteogenesis, mandibular reconstruction, bone regeneration

## Abstract

Distraction osteogenesis (DO) is a reconstruction method for repairing bone deficiencies in the oral and maxillofacial area. Manual DO techniques have shown the functionality of the DO method for bone tissue reconstruction. The DO method can improve treatment conditions, as well as the quality of the reconstructed bone, compared with conventional techniques. Recently, continuous DO devices have been proposed to enable an automatic DO process while using a continuous force for moving the bone segment (BS). Animal studies and clinical trials have shown the successful application of continuous distractors in terms of improving DO factors, including rate and rhythm. The continuous DO technique can shorten the treatment time and enhance the quality of the regenerated tissue. However, the developed continuous distractors are yet to be used in human applications. In this study, by combining motor-driven and hydraulic techniques, a hybrid distractor is proposed. The hybrid distractor is capable of generating a continuous distraction force while controlling the position of the BS in a linear vector, with a high positioning accuracy. Results of modelling and experimental study revealed that the proposed hybrid distractor met all required factors for enabling a continuous DO procedure in humans. The proposed distractor is capable of eliminating the drawbacks of exiting techniques in terms of generating and transferring a controlled distraction force to the BS. The wireless control, as well as the small size of the device, makes this device a suitable solution for use in the reconstruction of bone defects in the maxillofacial area in humans.

## 1. Introduction

Distraction osteogenesis (DO) is a biological process which is used to reconstruct skeletal defects or deformities by producing new bone and overlying soft tissue [1,2,3]. DO has played a major role in maxillofacial reconstruction applications (MRA) and has produced superior results when compared to conventional reconstruction techniques such as osteoconduction/osteoinduction, autologous bone grafts, and allograft implantation. The application of the DO technique in MRA results in reduced complications, such as shorter hospitalisation periods, no donor site morbidity, and reduced surgical complications [2,4,5].

**Figure 1:**
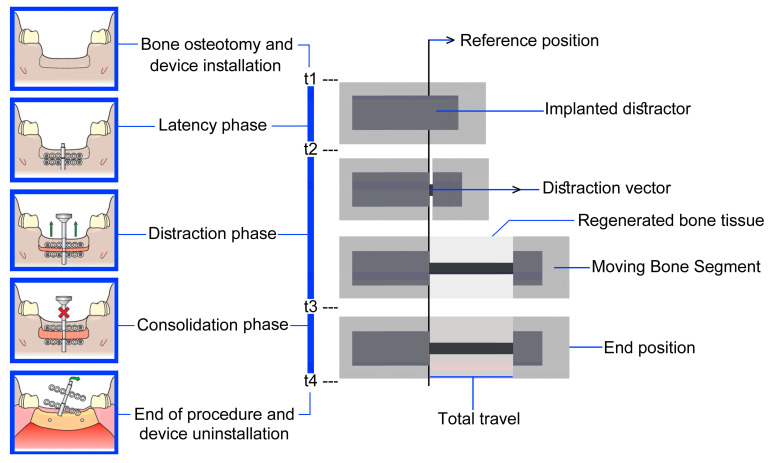
The standard DO method in MRA [7].

The DO technique has been researched continuously, beginning in 1987 with Ilizarov [6], who developed the DO technique. As the DO technique has progressed, distractors have continually been developed and improved, to allow for an optimal DO device [7]. Manual distractors can either be applied intra- or extra-orally. The first manual extra-oral distractor was developed by Ilizarov [6,8,9], after which manual extra-oral devices showed a high success rate, and caused the wide application of manual distractors in MRA [10]. DO consists of four predominant phases, including osteotomy, the latency phase, the distraction phase, and the consolidation stage [2,11]. A typical DO procedure in MRA, using an intra-oral manual distractor, is illustrated in Figure 1. The treatment begins with the osteotomy of the bone and installation of the bone-borne distractor (t1), after which the latency period commences (t2). The latency phase can typically last between three and ten days, and is the period in which the surgical site is allowed to heal and pass into the reparative phase. The restoration of the bone is likely to be observed by means of callus tissue, which has formed around and between the segments of the bone. The DO device is inactive during this period. The distraction period (t2–t3) is the period in which the DO device is active, and traction is applied such that there is a formation of woven bone fibers forming parallel to the distraction force (DF). The final phase is the consolidation period. The consolidation period (t3–t4) is the period in which there is an occurrence of the mineralization of the newly formed bone, while the DO device is inactive, and no DF is applied. This phase tends to exist after a period of a few days to a few weeks, based on the reconstruction conditions. Finally, the intra-oral distractor is removed (t4) [2,4].

Upon the implementation of a manual distractor, the DF is required to occur due to manual activations by the operator. The extra-oral distractor raised concerns such as scar formation, infection, and patient discomfort, while requiring manual adjustment of the DF which left further room for error [7]. Intra-oral devices seem to be more suitable techniques for use in MRA. Although manual distractors have been used in MRA, the distraction process relies on manual adjustment of the length, presenting potential error in the length adjustment as well as a large amount of uncertainty related to the DF. Manual distractors thus present multiple limitations including unstable movement, low distraction rate (DR) and distraction rhythm, long treatment time, scar formation, and painful distraction for the patient [12,13,14].

### Automatic Continuous Distractors

Studies revealed that the application of quasi-continuous DO techniques, with higher distraction rhythms, have produced results with higher distraction accuracies, and improved bone generation and consolidation phases. Due to the above-mentioned advantages, researchers have focused on the development of continuous distractors [12,15]. Upon the implementation of an automatic continuous distraction osteogenesis (ACDO) device, an automatic method is used, in which a continuous force is produced to move the bone segment (BS) towards the correct position in a linear vector [7]. Due to the increased DR observed upon the implementation of an ACDO distractor, reduced treatment periods, as well as patient discomfort and pain, could be observed, whilst improving the outcome of the treatment [5,16]. The viability of a continuous DO method has been proved in recent animal studies which have proved that the application of an ACDO solution leads to superior treatment results upon comparison with manual treatment methods. Additionally, it has been indicated that the application of a continuous DF could significantly increase the rate and the rhythm of distraction, and improve the tissue regeneration/healing mechanisms [17,18,19,20,21].

ACDO is a novel method, which has proved to meet the requirements of the standard DO protocol but is yet to be applied in human MRA, due to complications/limitations which are present within the existing methods, including the method of force generation and the size of intra-oral device [7]. In recently performed studies, motor-driven and hydraulic ACDO devices have shown promise, with motor-driven systems showing fewer complications and errors upon application. An accurate and precise DF could be executed using motor-driven devices, with a positioning accuracy down to 0.01 micrometer [22]. 

Different limitations are associated with the method of force generation in the developed hydraulic and motor-driven techniques. A pick force is generated at the commencement of the DF during DO performed with a hydraulic device, resulting in damaged hard/soft tissue during the distraction of the BS. Further complications during DO performed with a hydraulic distractor could result due to the large size of the extracorporeal and intra-oral parts of the device. Hydraulic systems also raise concern in the area of power consumption. Alternatively, developed motor-driven devices caused concern upon transferring the DF to the implanted part of the device [7]. Different mechanisms were used to transfer the generated continuous force to the moving BS. However, there is still a gap between the developed mechanisms and an ideal continuous distractor for use in human MRA.

The purpose of this study is the design and development of a novel continuous distractor to be used in human MRA. The proposed system is a hybrid mechanism, which combines the hydraulic and motor-driven techniques for generating and transferring the DF to the BS. In the proposed system, a controlled and smooth pushing force is generated, using a motor-driven mechanism. The generated force pushes the piston of a miniature hydraulic cylinder, and subsequently, the generated DF is transferred to the BS, using a hydraulic transition mechanism. By combining the motor driven and hydraulic techniques, the existing limitations in the developed methods have been met. The proposed hybrid distractor can generate a controlled continuous DF while eliminating the complications of force generation/transition mechanisms. The proposed device can be used as an ideal solution for MRA in humans.

## 2. Material and Methods

The designed hybrid distractor works based on a combined technique, by using motor-driven and hydraulic systems. The designed mechanism can generate a smooth and precise DF and apply it to the BS, to move the BS in a linear distraction vector. The hybrid distractor consists of three main parts, including a wireless control unit, an extracorporeal hybrid distractor, and an intra-oral mechanical part. By using the proposed system, a smooth and continuous DF is generated using a motor-driven mechanism. Subsequently, by using a hydraulic system, the generated DF is transferred to the mechanical part installed on the distraction zone.

### 2.1. Control System

**Figure 2:**
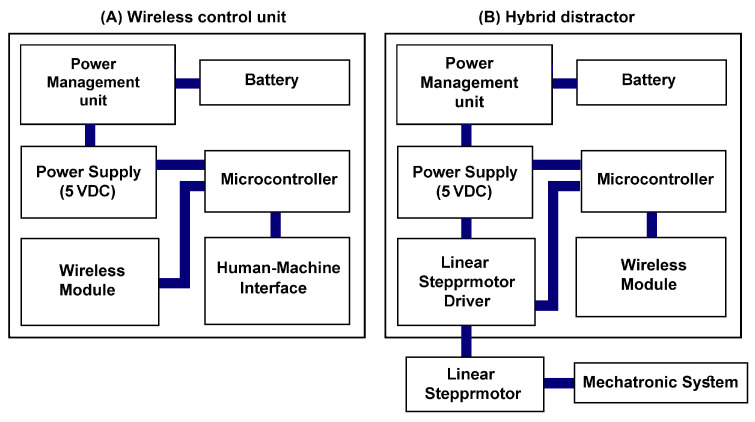
The block diagram of the system.

The control system of the device consists of two control units that communicate wirelessly. Figure 2 presents the block diagram of working principles of the hybrid distractor. In the designed control system, a wireless control unit is used to control the performance of the hybrid distractor. By using the wireless control unit, controlling the performance of the hybrid distractor and setting the distraction parameters, including DR, the distraction rhythm, and the length of distraction, is possible. The data set is transmitted to the control unit of the hybrid distractor using wireless communication. The control unit of the hybrid distractor, controls the performance of the hybrid mechanism and runs the device using the set working parameters. By using a high-precision control system, the stepper motor driver runs the stepper motor while generating a smooth and continuous pushing force. At the end, by using a force transition mechanism, the pushing force is transmitted to the intra-oral bone-borne distractor installed on the defected zone. The transmitted force, called DF, can push the BS and move it in a linear vector.

**Figure 3:**
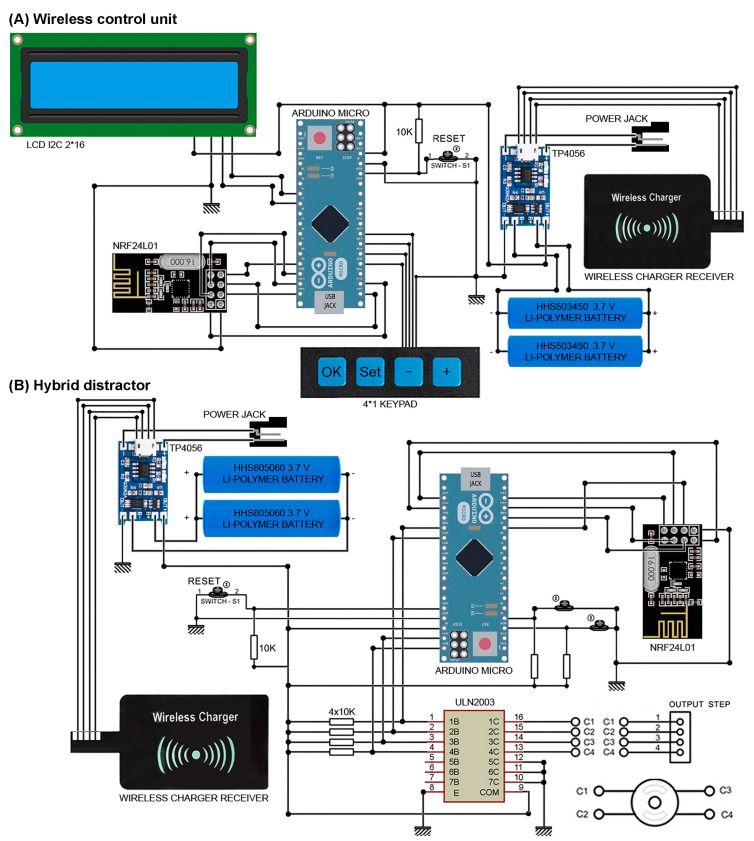
The detailed design of the control system: (**A**) Wireless control unit and (**B**) Hybrid distractor.

Figure 3 presents the detailed design of the control system of the hybrid distractor. In the wireless control unit, a liquid crystal display (LCD) and a membrane 1 × 4 keypad are used. The wireless control unit can be used to set, monitor, and modify the distraction parameters during the reconstruction process. In the wireless control unit, an Arduino MICRO A000053 development MCU board is used to control the performance of the wireless control unit, and to communicate with the hybrid distractor. In this unit, a wireless module, NRF24L01, is connected to the microcontroller to enable a wireless communication between the wireless control unit and the control system of the hybrid distractor. In addition, a power management system, the TP4056 battery charge controller, is connected to the supply of the system to supply 5 VDC and recharge the HHS503450 3.7 V lithium-polymer rechargeable batteries. In addition, a wireless charger receiver is implemented within the power management system for enabling the wireless charging of the batteries. The control unit transmits the data, including the set distraction parameters, to the hybrid distractor. After the input data is set, the operation can be run, using the wireless control unit.

The extracorporeal hybrid distractor is the main part of the device. The hybrid distractor consists of a control unit, a miniature mechatronic system, and a rechargeable battery system. In the hybrid distractor, an Arduino MICRO A000053 development MCU board is implemented to receive and analyse the set data, and to control the distraction process. A wireless NRF24L01 module is connected to the microcontroller for enabling communication with the wireless control unit, which allows wireless communication and data transmission between the implemented microcontrollers. By connecting a TP4056 battery charge controller to the system, it is possible to provide 5 VDC for running the control system, drive the linear mechanism, and charge the battery is possible. In addition, a wireless charger receiver is implemented within the power management system, for enabling wireless charging of the batteries. Two HHS605060 3.7 V lithium-polymer rechargeable battery cells are implemented within the power management unit to supply the device during the distraction process. In addition, a ULN2003 stepper motor driver is implemented within the system, for driving the stepper motor.

### 2.2. Hybrid Mechanism

The hybrid mechanism consists of a miniature motor-driven linear system combined with a hydraulic cylinder and fluid-based transition system. The designed mechanism can generate a continuous pushing force, and transfer the generated force via a flexible transition system to the intra-oral part. Subsequently, the transferred DF pushes the BS smoothly in a linear path towards the desired position. 

**Figure 4:**
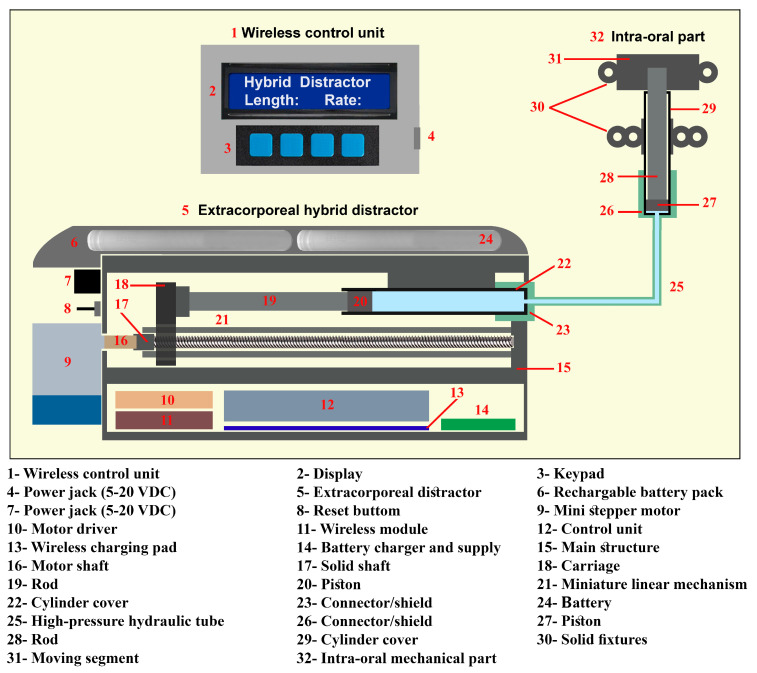
The schematic design of the hybrid distractor.

Figure 4 illustrates the schematic of the proposed hybrid distractor. In the mechatronics system, a 28BYJ-48 hybrid mini stepper motor and gear-box is used. This stepper motor has a step angle of 5.625 and a shaft stride angle of 0.088, and a gear box ratio of 1/64. The control system can drive the stepper motor in micro-stepping mode. In line with the set working parameters, the microcontroller sends the control signals to the ULN2003 motor driver. The output pins of the motor driver are connected to the stepper motor phases. The stepper motor shaft is connected to the lead screw of the linear mechanism via a solid shaft coupling. In the linear mechanism, a lead screw of 4 mm diameter, with 1-mm lead, 1-mm pitch, and a length of 80 mm, is used. The above-mentioned method allows the rotation of the lead screw of the linear system, the translation of the rotation to the linear motion, and the movement of the carriage of the mechanism along a linear axis. By driving the hybrid stepper motor in micro-step driving mode (1/32), the angular position of the shaft can be controlled with a step angle of 0.176° and a shaft stride angle of 0.00275°. Therefore, the carriage of the system can be moved along a linear axis, with a maximum positioning accuracy of 7.6 nm/step in the micro-step driving mode.

As presented in Figure 4, the carriage of the linear mechanism (18) is fixed to the rod of the hydraulic cylinder. The generated DF pushes the carriage precisely along a linear axis. Thus, the movement of the carriage is transferred to the hydraulic part of the mechatronic system. Subsequently, the generated DF pushes the hydraulic fluid inside the cylinder. A high-pressure hydraulic tube is connected to the output of the cylinder. The other side of the transition tube is connected to a miniature hydraulic cylinder, which is used in the intra-oral mechanical part of the distractor. By driving the stepper motor, the DF is generated and the hydraulic fluid is pushed and transferred to the intra-oral bone-borne distractor, through the flexible high-pressure tube. 

By using the positioning accuracy of the linear mechanism, the positioning accuracy of the end effector, cylinder (27) and the moving segment of the mechanical distractor, can be calculated. Theoretically, the linear system can generate a pushing force of 35 N with a movement accuracy of 7.6 nm in micro-stepping drive mode. The cylinder (20) has a diameter of 3.5 mm, and the cylinder (27) has a diameter of 3 mm. Therefore, in the case where 35 N pushing force is applied to cylinder (20) and moves it for 7.6 nm, the cylinder (27) is pushed with a pushing force of 25.5 N and moved for 10.5 nm.

**Figure 5:**
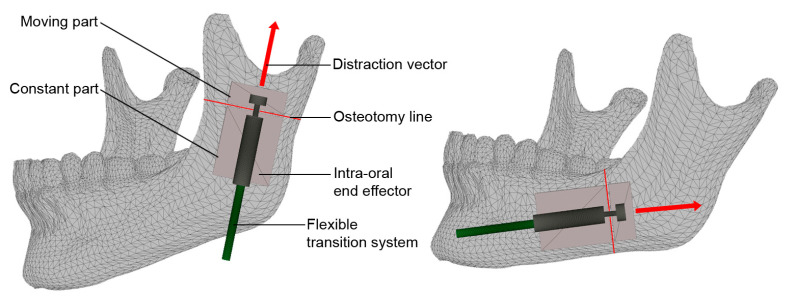
The application of the intra-oral bone-borne end effector.

**Figure 6:**
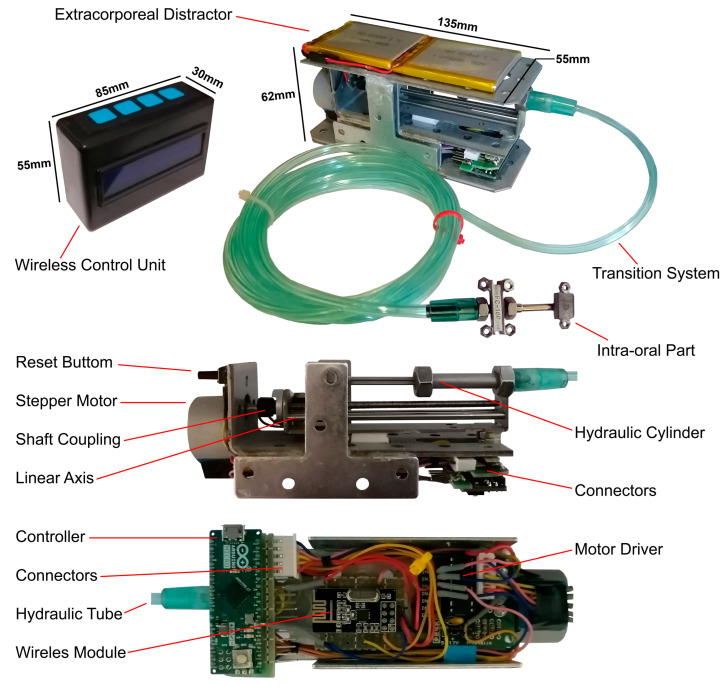
The first prototype of the hybrid distractor.

Figure 5 illustrates the application of the intra-oral bone-borne end effector as well as the method of bone distraction in a linear distraction vector. The body of the cylinder (29) is fixed to the main bone part via mechanical fixtures (30). The moving cylinder is connected to the moving part of the mechanical structure (31) by using a rod (28). The transferred DF pushes the cylinder (27) along a linear axis. Therefore, the BS can be moved in a linear vector towards the desired position, using the generated continuous DF. After the design of the device, the hybrid distractor was developed. Figure 6 presents the first prototype of the device which is used in the experimental verification of this study.

### 2.3. Power Consumption of the System

In the designed system, battery cells and power management units are used to supply the hybrid distractor and the wireless control unit. Using the rechargeable battery system makes the device portable. It is important to select suitable battery cells with enough capacity, so that they can supply the device with the required power specifications [23]. Therefore, according to the specifications of the components that are used in the device, the power consumption of the system was calculated. Subsequently, suitable batteries were selected and implemented within the rechargeable battery system of the device. 

**Figure 7:**
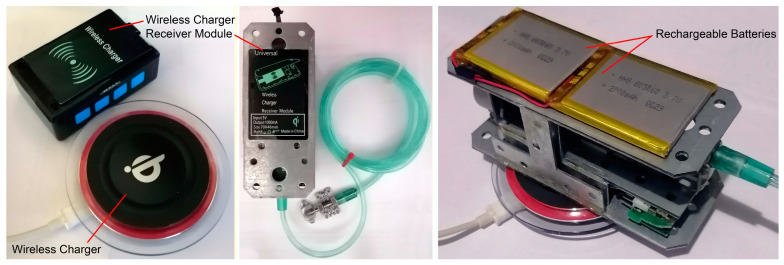
The wireless power charging system.

Figure 7 presents the wireless power charging system of the device. In the wireless control unit, a wireless module, an LCD, a power management module, and a microcontroller are used. According to the specifications of the mentioned components, the wireless control unit has a power consumption of 185 mAh. In this unit, two HHS503450 lithium-polymer rechargeable batteries are connected in a series configuration, to supply the unit with a total capacity of 7.4 W. Therefore, this the battery cell is capable of supplying the wireless control unit for 8 h before the need of a recharge. 

In the same way, the power consumption of the hybrid distractor can be calculated. In the hybrid distractor, a microcontroller, a wireless module, a power management module, a motor driver, and a hybrid stepper motor are used. According to the power consumption of the mentioned components, the hybrid distractor has a power consumption of 266 mAh. In the hybrid distractor, two HHS605060 lithium-polymer battery cells are connected in a series configuration to supply the hybrid distractor with a total capacity of 14.8 W. Therefore, the battery system is capable of supplying the hybrid distractor for 11 h before the need of a recharge. In both the wireless control unit and the extracorporeal hybrid distractor, a wireless charger receiver module is connected to the power management unit to enable wireless charging of the battery cells. 

### 2.4. Modeling of the Control System

**Figure 8:**
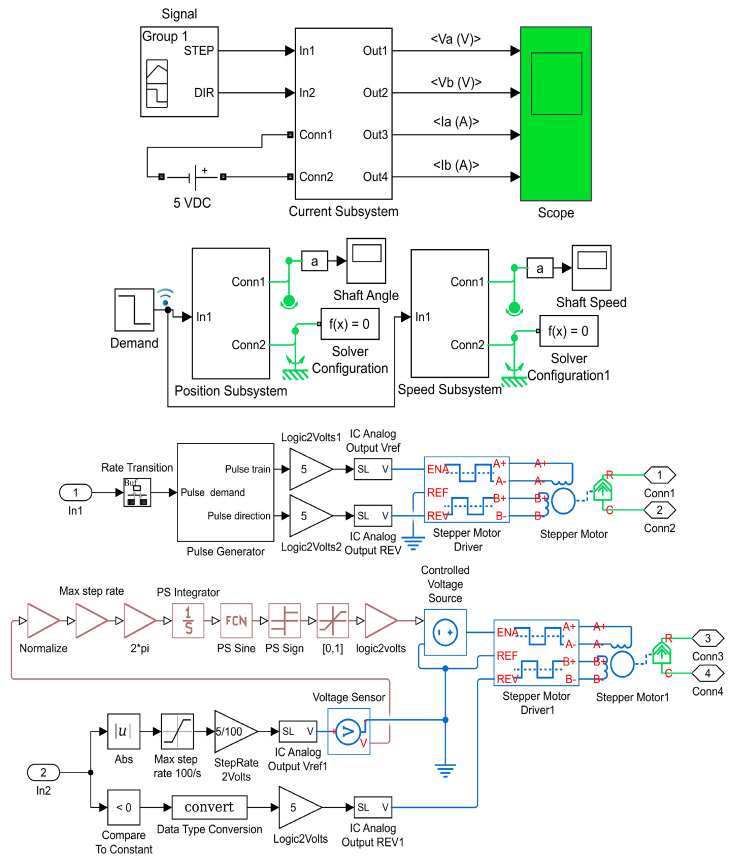
The modelling of the control system.

The control system of the hybrid distractor should be capable of controlling and driving the implemented hybrid stepper motor. For this purpose, a high-precision linear control technique [24,25] is used in the design of the control system. By using the differential equations of the stepper motor [26], as well as the specifications of the hybrid stepper motor, the control system was modelled in MATLAB/SIMULINK software. Figure 8 presents the modelled control system.

### 2.5. Experimental Study

Figure 6 presents the first prototype of the hybrid DO device. A series of experimental tests were conducted, to evaluate the performance of the developed hybrid distractor. It is important that the developed distractor be able to generate the desired DF with a high positioning accuracy.

**Figure 9:**
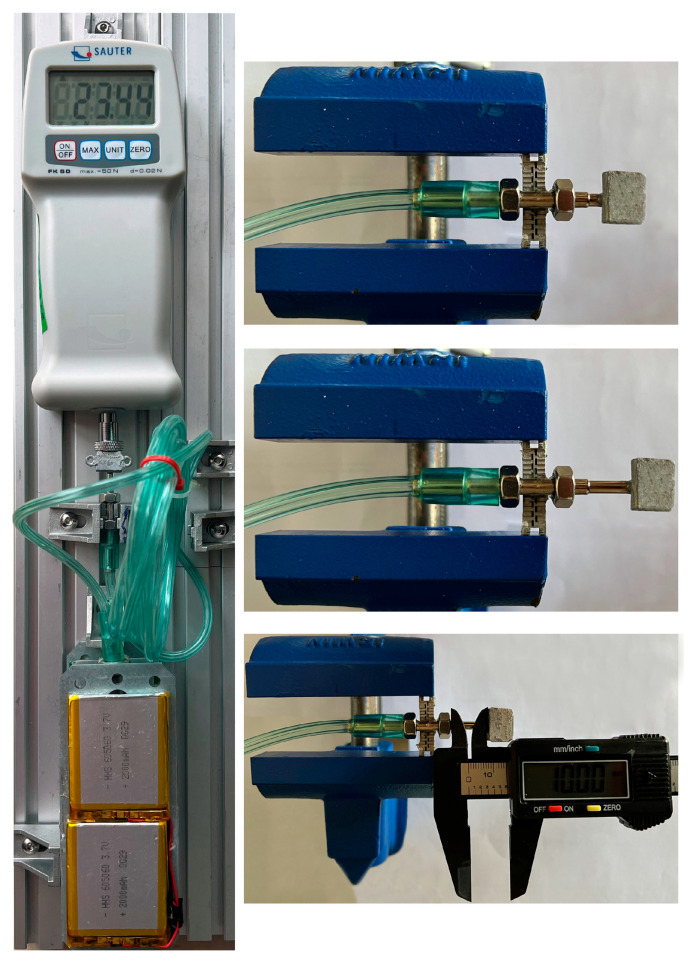
Measurement of the generated force and the linear positioning accuracy.

In the first phase of the experimental study, the amount of the generated DF was evaluated. For this purpose, a digital force gauge, Sauter FK-50, was interacted with the mechanical part of the distractor, for measuring the generated pushing force (DF) in the end effector. Figure 9 presents the condition of the force measurement test. 

In the second phase of the experimental study, the device was fixed while working under different conditions, to evaluate the performance of the device in terms of accurate positioning and to execute a standard distraction process, with the desired working factors. In this experiment, an RS PRO digital calliper, with a resolution and precision of 0.01 mm, was used, for the measurement of the total travel of the end effector. Figure 9 presents the condition of the linear positioning test. According to the literature, as well as the results of performed experimental studies, it can be deduced that in a standard DO process using ACDO devices, the following parameters are usually used: DR: 1–3 mm/day. Distraction length: 10–30 mm. DF: 20–35 N. Treatment period: 7–10 days [5,7,13]. Therefore, in the experimental study, different DO processes were performed using various working parameters, to evaluate the performance of the device under different treatment conditions. Table 1 presents the set working parameters used in the experimental tests.

## 3. Results and Discussion

**Figure 10:**
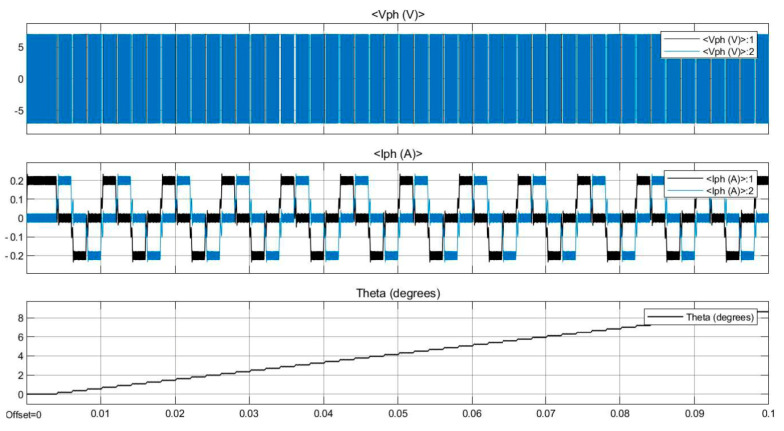
Simulation results.

The control system can generate control signals and drive the stepper motor of the linear mechanism using an open-loop control system. Simulation of the modelled control system was run for 0.1 s and graphical results were produced. The simulation results, including four waveforms, are presented in Figure 10, where, V_1_ and V_2_ are the voltages in the stepper motor phases, I_1_ and I_2_ are the currents in the stepper motor phases, and theta is the shaft position of the stepper motor. It can be seen that the voltage and current waveforms in phase A and B are 90° displaced, and agree with theoretical equations. In addition, the shaft angle and shaft speed waveforms show that the stepper motor can execute a soft rotation with low noise and ripple. The positioning of the rotor as well as the rotor speed are well controlled. The simulation results show that the control system can drive the stepper motor with the desired working factors. It can be deduced from the simulation results that the designed control system used in this device, work well in different conditions. Driving the stepper motor in micro-stepping drive mode could provide a smooth and controlled rotation of the motor shaft, which secures a smooth and continuous force generation.

In the experimental study, the generated DF and the executed linear positioning of the distractor were evaluated, as illustrated in Figure 9. For the measurement of the generated force, the extracorporeal hybrid distractor and the intra-oral part were fixed while the moving part of the end effector was pushing the force gauge. The force measurement test showed that the hybrid distractor can generate a continuous pushing force (DF) of 23.44 N. According to the literature, as well as the standard DO protocol, the generated pushing force is sufficient for performing a successful DO process in MRA. It is also possible to decrease the diameter of the cylinder (20) to increase the generated DF in the cylinder (27) in specific MRA, where a higher amount of DF is required.

**Figure 11:**
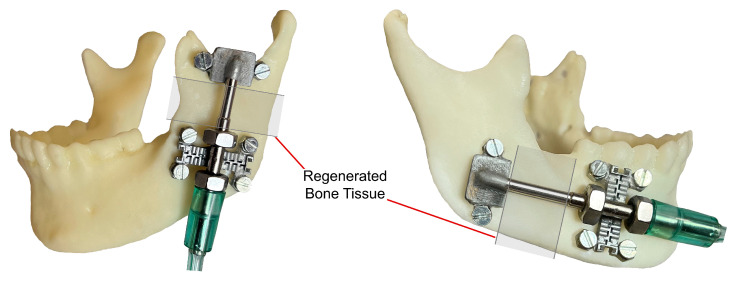
Illustration of the application of intra-oral bone-borne end effector in MRA.

In another experiment, the linear positioning of the system was evaluated. For this purpose, the device was operated while performing distraction with various working factors, as presented in Table 1. In all test conditions, the positioning of the linear mechanism was easily achieved, without any error during the performance of the hybrid distractor. The hybrid distractor is capable of executing a smooth and stable linear motion with high positioning accuracy. The results show that the linear system has a positioning accuracy of 0.01 µm, with a positioning error rate of 0.2% in achieving the set distraction length. The developed DO device can perform distraction with standard factors. The system is capable of generating linear movement with high positioning accuracy while achieving the desired length under different working conditions, with high accuracy and repeatability. Figure 11 illustrates the application of the developed hybrid distractor in MRA.

An important aspect in the development of automatic distractors is the power consumption of the system and the capabilities of the rechargeable battery system of the device. It is important to have a mobile device with good charging capacity, including the duration that the device can work using batteries. In this system, two HHS605060 lithium-polymer batteries, with a total battery capacity of 7.4 Wh and a nominal voltage of 3.7 V, are connected in series, while providing the power for running the device with a total capacity of 14.8 Wh. According to the specifications of the hybrid distractor, it has a power consumption of 1.33 Wh. Therefore, the battery system is capable of supplying the system with a voltage rate of 5 VDC and a current of 266 mA, for 11 h before the need of a recharge. In addition, in the wireless control unit, using two 3.7 V 1000 mAh battery in series, with a total capacity of 7.4 Wh, can provide the required energy for running the wireless control unit, which has a power consumption of 0.9 Wh, for 8 h before the need of a recharge. The device has a continuous performance from the start of the distraction process to the end of the activation phase. There is no need to stop or turn off the device for recharging the batteries. It is possible to charge the batteries using a 5 VDC power charger (wired) or wireless charger, while the device is active. 

In the proposed method, the required pushing force for moving the BS is generated in the extracorporeal distractor and transmitted to the intra-oral part of the device via a miniature flexible tube. This specific hydraulic-based force transition mechanism is used to eliminate the need for a power source in the intra-oral part of the device and to decrease the size of the intra-oral distractor. In addition, electromechanical fault/error is unavoidable, and could potentially happen during the performance of any medical device. In this case, the extra-corporeal electromechanical part of the device could be easily replaced, without affecting the reconstruction process.

DO is an emerging technique in MRA. Recently, continuous devices, using motor-based and hydraulic systems, have been developed to enable continuous DO in the maxillofacial area. The motor-based systems have shown good functionality in generating smooth and continuous DF for performing DO; however, there are limitations in transferring the generated DF to the distraction zone. Hydraulic systems have shown limitations in producing a smooth and continuous DF [7,27,28]. The proposed hybrid distractor can eliminate the existing limitations of the both motor-based and hydraulic systems by using a hybrid mechanism for producing the DF. The developed hybrid DO device is capable of performing a standard DO process with high positioning accuracy and repeatability while producing a smooth and continuous DF for moving the BS.

## 4. Conclusions

The DO technique is an effective solution for the reconstruction of bone defects in MRA. Recently, different continuous distractors have been developed for use in different DO treatments. However, there are complications associated with existing devices, and they are yet to be commercialized and used in human applications.

In this study, a hybrid distractor is developed. By combining motor-driven and hydraulic mechanisms, a hybrid mechanism is developed, to generate a continuous DF. The results of the experimental study, as well as the results of the simulation, revealed that the hybrid distractor meets all requirements for performing a standard DO in MRA. The developed hybrid mechanism eliminates the disadvantages of motor-driven and hydraulic distractors, while providing a controlled and precise movement of the BS. The intra-oral part of the system has a small size, which is suitable for use in human MRA.

The results of the simulation show that the designed control system is functional and able to execute controlled linear movement with high positioning accuracy while producing a controlled continuous pushing force. The results of the experimental tests revealed that the hybrid distractor can generate a continuous DF of 23.44 N, with a positioning accuracy of 0.01 µm and a positioning error rate of less than 0.2%. The rechargeable battery system makes the device portable, while a wireless control pad can control the performance of the device and set/monitor the distraction parameters. The developed continuous distractor is an ideal solution for use in MRA.

## 5. Patents

A patent was produced from the research work reported in this manuscript. The method of continuous force generation and the force transition mechanism are under the protection of Taiwan’s Intellectual Property Office, as an invention patent, with publication/patent number M604182, application number 109209126, entitled: “Hybrid traction device for oral and maxillofacial reconstruction”.

## Figures and Tables

**Table 1:** The set working parameters in the positioning test and the obtained results.

Test	DR (mm/day)	Desired Distraction Length (mm)	Distraction Time (Hour)	Repeat Cycle	Mean Measured Distraction Length (mm)	Mean Positioning Error (µm)	Positioning Error Rate (%)
T.1	1	5	120	2	5.01	10	0.2
T.2	2	10	120	5	10.01	10	0.1
T.3	2	20	240	2	20.01	10	0.05
T.4	3	10	80	5	10.01	10	0.1
T.5	3	20	160	2	20.02	20	0.1
T.6	4	10	60	5	10.02	20	0.2
T.7	4	20	120	2	20.01	10	0.05

## Data Availability

The research data related to this work are included within the manuscript. For more information on the data, contact the corresponding authors.
